# Supplementary material for: IFT cargo and motors associate sequentially with IFT trains to enter cilia of C. elegans
Source: Nat Commun. 2024 Apr 24;15:3456. doi: 10.1038/s41467-024-47807-2 (PMC11043347; doi:10.1038/s41467-024-47807-2)
Supplement: Supplementary file 1 — Supplementary Information [file 41467_2024_47807_MOESM1_ESM.pdf]

Supplementary information for the manuscript:

IFT cargo and motors associate sequentially with IFT trains to enter cilia of *C. elegans*

Aniruddha Mitra[1,2], Elizaveta Loseva[1] and Erwin J.G. Peterman [1,*]

[1] Department of Physics and Astronomy and LaserLaB, Vrije Universiteit Amsterdam, Netherlands
[2] Present address: Cell Biology, Neurobiology and Biophysics, Department of Biology, Faculty of Science, Utrecht University, The Netherlands.
* Correspondence: e.j.g.peterman@vu.nl

Supplementary Figures:

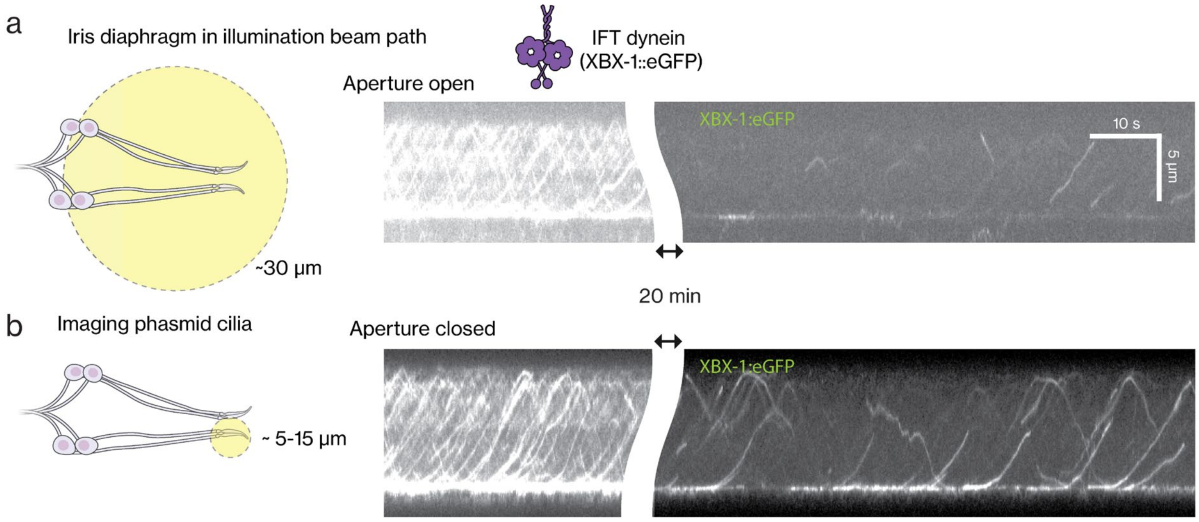

Supplementary Fig. 1: SWIM allows long-duration single-molecule imaging of IFT-dynein (XBX-1::eGFP) entering the cilia. (a) Single-molecule imaging of IFT-dynein (XBX-1::eGFP), using the whole excitation beam (diameter ~30 µm; aperture open) to illuminate the sample worm (illustration in left panel). Representative kymograph of IFT-dynein along an imaged cilia-pair, initially shows a large pool of IFT-dynein moving back and forth in the cilia, which bleaches almost completely over time, as seen in the segment of the kymograph after 20 min of imaging. **(b)** Single-molecule imaging of IFT-dynein, using SWIM, exciting only a small region (diameter ~5-15 µm; aperture closed; illustration in left panel). As in A, representative kymograph of IFT-dynein along an imaged cilia pair, initially shows a large pool of IFT-dynein in the cilia. After bleaching of the pool of IFT-dynein in the cilia, one can still observe unbleached IFT-dynein molecules that are continuously entering the small excited region over long duration. Since the edge of the excited region is placed near the ciliary base, one can observe that it is rather busy, with individual IFT-dynein molecules continuously docking at the base and entering the cilia, even after 20 min of imaging. To reduce the frequency of observed single-molecule entry events, we usually illuminate also a part of the dendrite such that a higher fraction of fluorescing molecules photobleaches before docking at the ciliary base. Notably, the signal quality is far superior when using SWIM. Intensity has the same scaling in the kymographs in a and b. Supplementary Video 1 shows the movies from 20 min onwards, corresponding to the kymograph sections on the left in a and b.

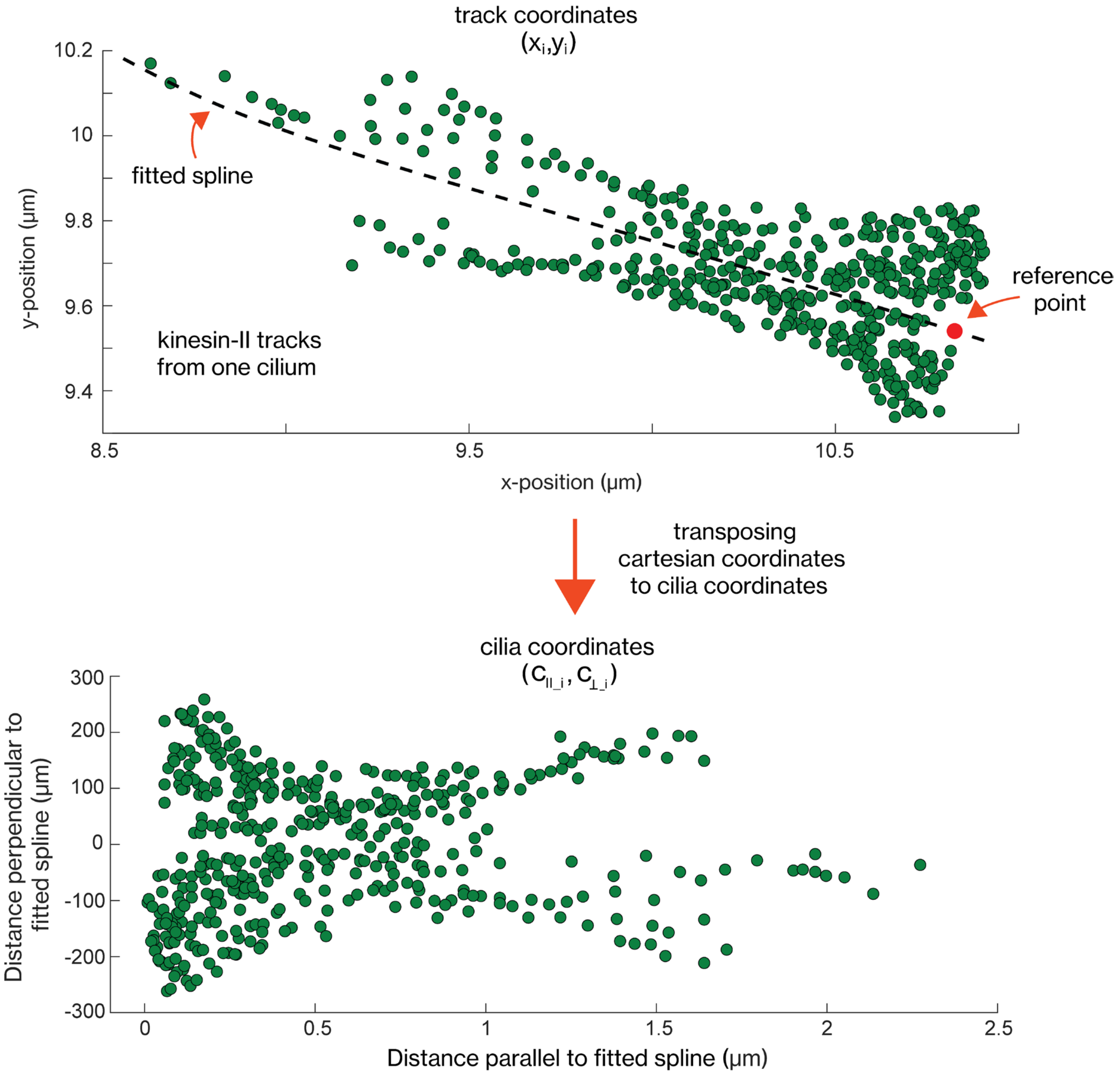

Supplementary Fig. 2: Transposing cartesian coordinates of single-molecule tracks to general cilia coordinates. Upper panel: x-y coordinates of all tracked kinesin-II entry events (476 single-molecule localizations from 20 tracks) in an imaged cilium. The single-molecule localizations provide the structure near the ciliary base, making it possible to draw a spline (dotted black line) roughly along the longitudinal axis of the cilium and select a reference point at the ciliary base. The cartesian coordinates of each single-molecule localization (x_i, y_i) can be transposed to distance perpendicular to the spline ($c_{\perp i}$) and distance from the reference point ($c_{\parallel i}$), referred to as cilia coordinates. Lower panel: Single-molecule localizations, plotted in cartesian coordinates in the upper panel, replotted in cilia coordinates.

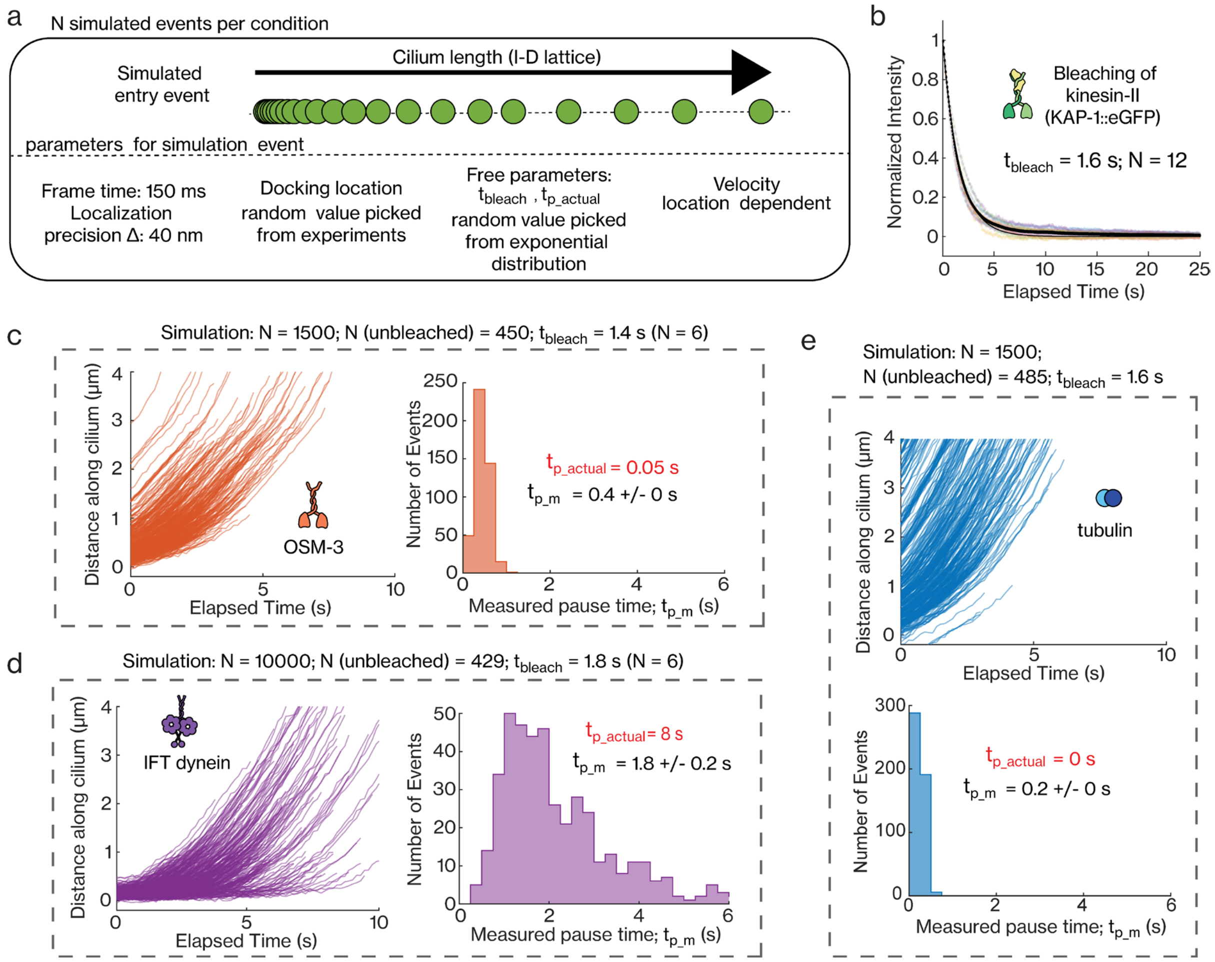

Supplementary Fig. 3: Numerical simulations to estimate actual pause time of single-molecule tracks entering cilia. (a) Scheme of the numerical simulation. Each simulated molecule is designated a bleach time (t_b) and 'actual' pause time (t_p), randomly picked from exponential distributions with rate parameters t_{bleach} (estimated from experiments) and t_{p_actual} (free parameter), respectively. The molecule docks along a 1D cilium lattice, with the docking location randomly selected from experimentally measure docking locations. After every time interval, the molecule either stays in the same location (elapsed time $t < t_b \,\&\, t_p$), moves forward ($t < t_b \,\&\, t > t_p$) with a location dependent velocity (obtained from experiments) or beaches ($t \geq t_b$; end of event). N is the number of events for a given condition, frame time is 150 ms and the localization precision is 40 nm. **(b)** Exponential decay of the kinesin-II intensity over time, upon exposure to high intensity 491 nm laser (number of cilia N = 12). The exponential fit (black line) provides a characteristic t_{bleach} = 1.6 s. **(c)** Distance-time plots of simulated OSM-3 events (N = 1500, N[unbleached] = 450) and histogram of measured pause time (average pause time t_{p_m} = 0.4 $\pm$ 0 s), for t_{p_actual} = 0.05 s. t_{bleach} is 1.4 s (N = 6). **(d)** Distance-time plots of simulated IFT-dynein events (N = 10000, N[unbleached] = 429) and histogram of measured pause time (average pause time t_{p_m} = 1.8 $\pm$ 0.2 s), for t_{p_actual} = 8 s. t_{bleach} is 1.8 s (N = 6). **(e)** Distance-time plots of simulated tubulin events (N = 1500, N[unbleached] = 485) and histogram of measured pause time (average pause time t_{p_m} = 0.2 $\pm$ 0 s), for t_{p_actual} = 0 s. t_{bleach} is 1.6 s. Average value and error are estimated using bootstrapping (see Methods).

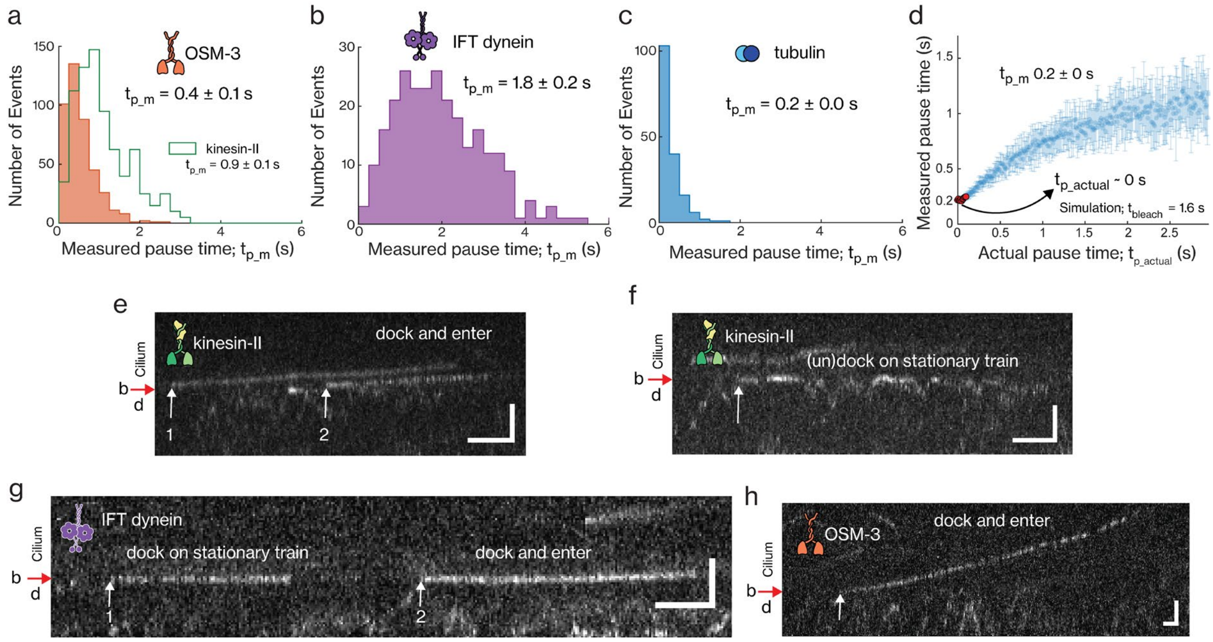

Supplementary Fig. 4: More details on dynamics of individual OSM-3, IFT-dynein and tubulin molecules entering cilia. (a-c) Histogram of measured pause times, t_{p_m}, for OSM-3 (average pause time is 0.4 $\pm$ 0.1 s), IFT-dynein (average pause time is 1.8 $\pm$ 0.2 s) and tubulin (average pause time is 0.2 $\pm$ 0 s). The distribution of measured pause times of kinesin-II in wild type is overlayed (green line) in (a) for reference. **(d)** Distribution of the measured pause times, t_{p_m}, with respect to actual pause times, t_{p_actual}, for tubulin, obtained from numerical simulations (using t_{bleach} = 1.6 s). Each point represents the average t_{p_m} for a single simulated experiment of 1500 events (example simulation in Supplementary Fig. 3e), varying t_{p_actual} by steps of 0.01 s. Simulated experiments yielding t_{p_m} 0.2 $\pm$ 0 s (similar to experimental value; Supplementary Fig. 4c) are highlighted in red, indicating that t_{p_actual} is 0 s. **(e-h)** Example kymographs of kinesin-II, IFT-dynein and OSM-3 imaged at a fast acquisition rate (see Supplementary Video 3). Kinesin-II and IFT-dynein were imaged at ~60 fps and OSM-3 was imaged at ~31 fps. White arrows indicate the point where an individual molecule docks (for the first time) at the ciliary base. In all kymographs, the vertical scale bar is 2 µm and the horizontal scale bar is 0.5 s. **(e)** Kymograph displaying two kinesin-II entry events docking on IFT trains at the ciliary base from a diffusive pool in the PCMC, with molecule 1 moving unidirectionally into the cilium and molecule 2 switching to short diffusive bursts in between directional motion (see Supplementary Video 3a). **(f)** Kymograph displaying a diffusive kinesin-II molecule docking on and off from one (or more) stationary IFT trains at the ciliary base (also see Supplementary Video 3b). **(g)** Kymograph displaying two diffusing IFT-dynein molecules docking on stationary IFT trains at the ciliary base, with molecule 1 remaining static while molecule 2 unidirectionally entering the cilium after a pause (see Supplementary Video 3c). **(h)** Kymograph displaying an OSM-3 molecule docking on a moving IFT train, moving unidirectionally into the cilium (also see Supplementary Video 3d).

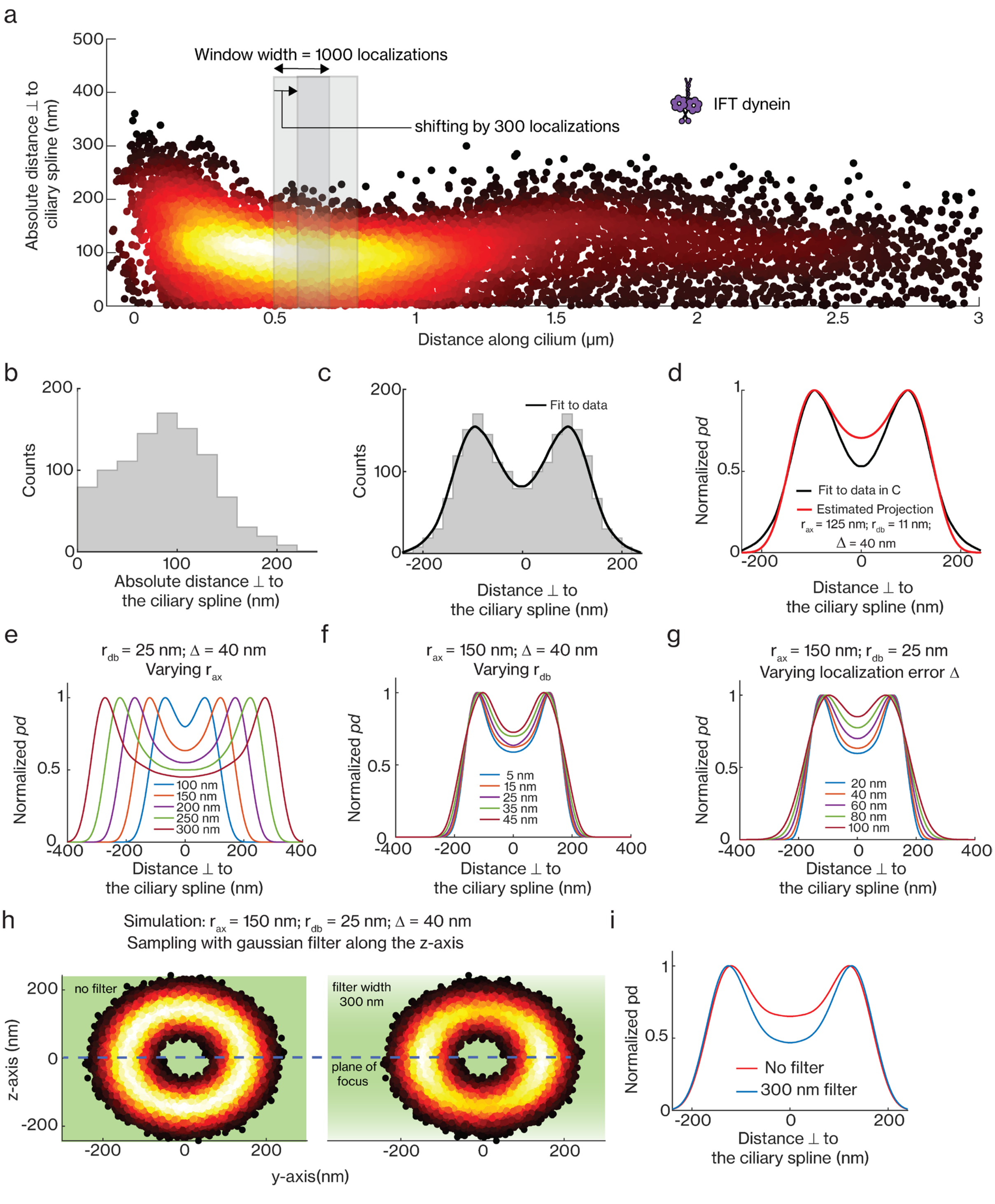

Supplementary Fig. 5: Estimating the underlying 3D distribution of the 2D projection map obtained from single-molecule localizations, using numerical simulations. (a) Super-resolution map of single-molecule localizations, plotting absolute distance perpendicular to the spline, obtained from 666 IFT-dynein tracks (N = 11314 moving localizations; same distribution as in right panel of Fig. 4a). The distribution of distance perpendicular to the spline is sampled every 1000 data points, moving every 300 data points for the next sampling. **(b)** Distribution of absolute distance of 1000 localizations, perpendicular to the spline, located between ~500-650 nm from the ciliary base (indicated with left shaded grey area in a). **(c)** Distribution of distance perpendicular to the spline, in b, mirrored across the spline and kernel density fit (black line) to the distribution. **(d)** Maximum likelihood estimation of kernel density fit in c (black), from a library of 2D projections (for a range of r_{ax} and r_{db}, obtained from numerical simulations, with N = 10000 and localization error Δ = 40 nm for each condition). The best estimate for this distribution (in red) is for r_{ax} = 125 nm, r_{db} = 11 nm. **(e-g)**

Distribution of 2D projections, obtained from numerical simulations (N = 10000 for each condition), varying r_{ax} (E; r_{db} = 25 nm and Δ = 40 nm; r_{ax} = 100 nm, 150 nm, 200 nm, 250 nm, 300 nm), r_{db} (F; r_{ax} = 150 nm and Δ = 40 nm; r_{db} = 5 nm, 15 nm, 25 nm, 35 nm, 45 nm) and Δ (G; r_{ax} = 150 nm and r_{db} = 25 nm; Δ = 20 nm, 40 nm, 60 nm, 80 nm, 100 nm). **(h)** Super-resolution maps of single-molecule localizations along the traverse section (yz-plane), simulated for r_{ax} = 150 nm, r_{db} = 25 nm and Δ = 40 nm (N = 10000), sampling all values (left panel; no filter) and sampling with Gaussian filter along the z-axis (right panel; 2σ = 300 nm for the Gaussian filter). **(i)** Distribution of 2D projections for simulated data shown in h, sampling with (blue) and without a Gaussian filter along the z-axis (red).

Supplementary text associated with Supplementary Fig. 5

To extract the underlying structure from the experimentally obtained localizations, we generated a library of simulated 1D projections (see Methods for simulation details), for varying r_{ax} and r_{db} (assuming Δ to be 40 nm). We then sampled distributions of IFT-dynein localizations (distance perpendicular to cilia spline), along the cilia length (Supplementary Fig. 5a; example distribution in Supplementary Fig. 5b-5c) and obtained the best estimate for r_{ax} and r_{db} from the 1D projection library, using maximum likelihood estimation. We found that while the peaks of the distribution, determined by r_{ax} (Supplementary Fig. 5e), can be accurately predicted, our simulations cannot fit the dip at the centre of the distribution observed in our experimental data (Supplementary Fig. 5d). While we do observe that varying r_{db} (Supplementary Fig. 5f) and Δ (Supplementary Fig. 5g) in our simulation alters the magnitude of the dip at the centre of the distribution, the dip in the experimental distribution is much sharper. One explanation for this is that in our experiments we under-sample localizations that are above (below) the plane of focus. Indeed, when we sample simulated localizations with a Gaussian filter along the z-axis (Supplementary Fig. 5h), we can replicate the distributions we observe experimentally. From this we conclude that due to experimental variables, such as localization error and under-sampling of localizations away from focus, we cannot accurately estimate the underlying 3D distributions from the 2D single-molecule localizations, in our experimental data.

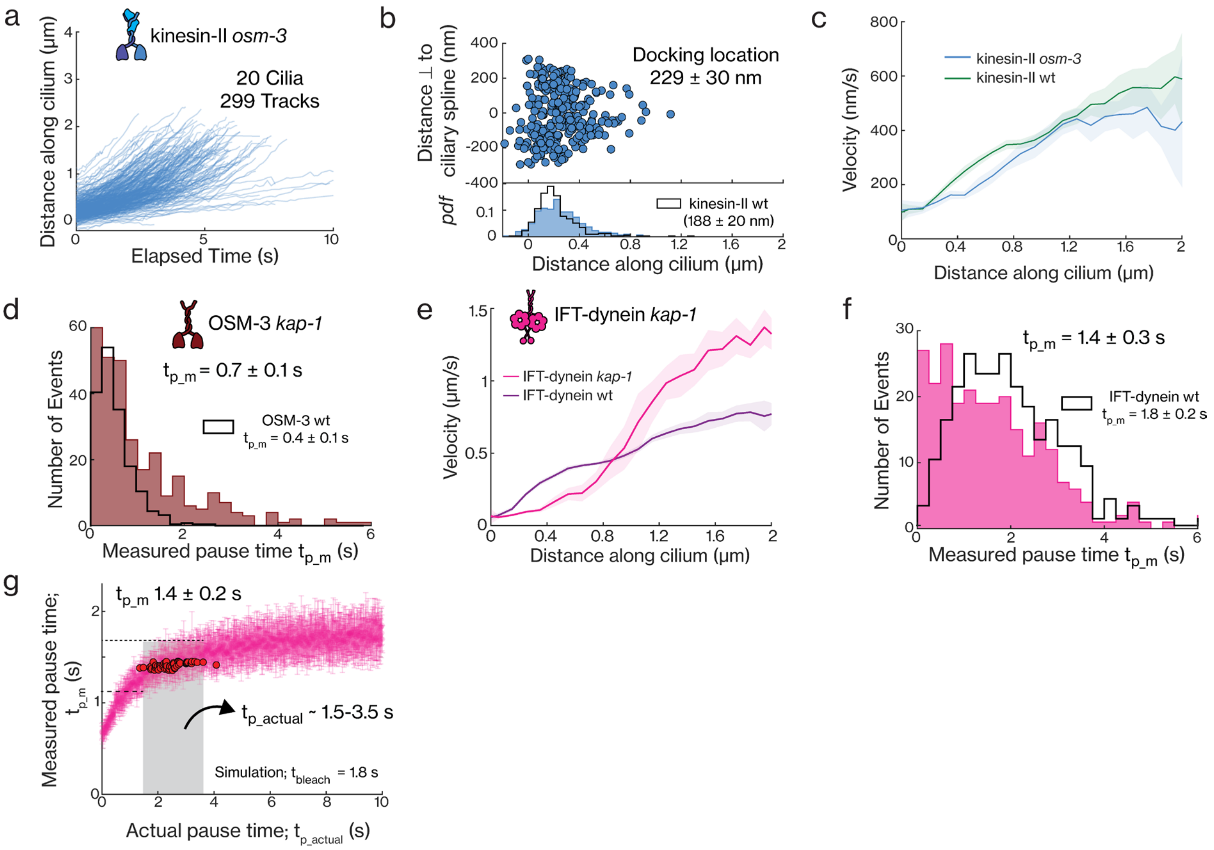

Supplementary Fig. 6: More details on entry dynamics of kinesin-II *osm-3,* OSM-3 *kap-1* and IFT-dynein *kap-1* molecules entering cilia. (a) Distance-time plots of kinesin-II *osm-3* (299 tracks from 20 cilia). **(b)** Distribution (upper panel) and histogram (lower panel) of docking locations of kinesin-II *osm-3* (average docking location is 229 $\pm$ 30nm). The distribution of docking location of kinesin-II in wild-type worms is overlayed in black. **(c)** Velocity distribution of kinesin-II along the ciliary length in wild-type (green) and *osm-3* mutant (blue) worms. Solid line is the binned average velocity and shaded area indicates the error. **(d)** Histogram of measured pause time, t_{p_m}, for OSM-3 *kap-1* (average pause time is 0.7 $\pm$ 0.1 s). The distribution of measured pause times of OSM-3 in wild type is overlayed (black line) for reference. **(e)** Velocity distribution of IFT-dynein along the ciliary length in wild-type (purple) and *kap-1* mutant (pink) worms. Solid line is the binned average velocity and shaded area indicates the error. **(f)** Histogram of measured pause time, t_{p_m}, for IFT-dynein *kap-1* (average pause time is 1.4 $\pm$ 0.3 s). The distribution of measured pause times of IFT-dynein in wild type is overlayed (black line) for reference. **(g)** Distribution of the measured pause time, t_{p_m}, with respect to actual pause time, t_{p_actual}, for IFT-dynein *kap-1* obtained from numerical simulations (using t_{p_bleach} = 1.8 s). Each point represents the average t_{p_m} for a single simulated experiment of 10000 events, varying t_{p_actual} by steps of 0.01 s. Simulated experiments yielding t_{p_m} 0.14 $\pm$ 0.2s are highlighted in red, indicating that t_{p_actual} is in the range 1.5-3.5 s. None of the simulations yield the experimentally obtained t_{p_m} (1.4 $\pm$ 0.3 s; 6f), hence the closest t_{p_m} is used to provide an estimate.

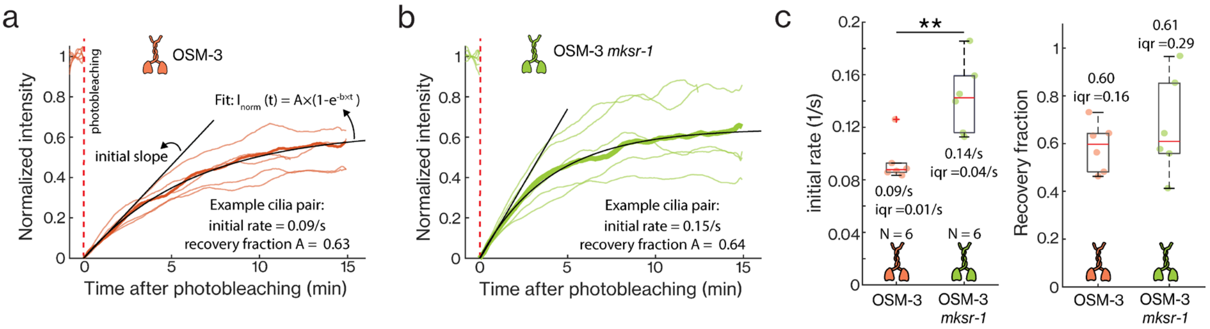

Supplementary Fig. 7: Fluorescence recovery after photobleaching (FRAP) for OSM-3 in wild-type cilia and *mksr-1* mutant cilia. (a) Normalized intensity of OSM-3 in wild-type cilium pairs plotted over time (N = 6), with the cilia photobleached at time t = 0. For an example cilium (bold orange line), the initial rate of recovery is 0.09/s and the recovery fraction is 0.63. The initial rate (black line) is obtained from a linear fit to the intensity measured in the first 10 time points after photobleaching, and the recovery fraction A is obtained from fitting $I_{norm}(t) = A \times (1 - e^{-b \times t})$ on the overall intensity over time (fit indicated with black line). **(b)** Normalized intensity of OSM-3 in *mksr-1* mutant plotted over time (N = 6), with the cilia photobleached at time t = 0. For an example cilium (bold green line), the initial rate of recovery is 0.15/s and the recovery fraction is 0.64. The fits to obtain the initial rate and recovery fraction are indicated with black lines. **(c)** Box plots of initial rate of recovery (left) and recovery fraction (right) obtained from FRAP curves of OSM-3 in wild type and OSM-3 *mksr-1*. The initial rate of recovery is significantly higher for OSM-3 *mksr-1* (p = 0.009; Mann-Whitney U test). The recovery fraction appears more variable for OSM-3 *mksr-1* but the distributions are not significantly different (p = 0.7; Mann-Whitney U test). The median value and the interquartile range (iqr) for each boxplot is indicated.

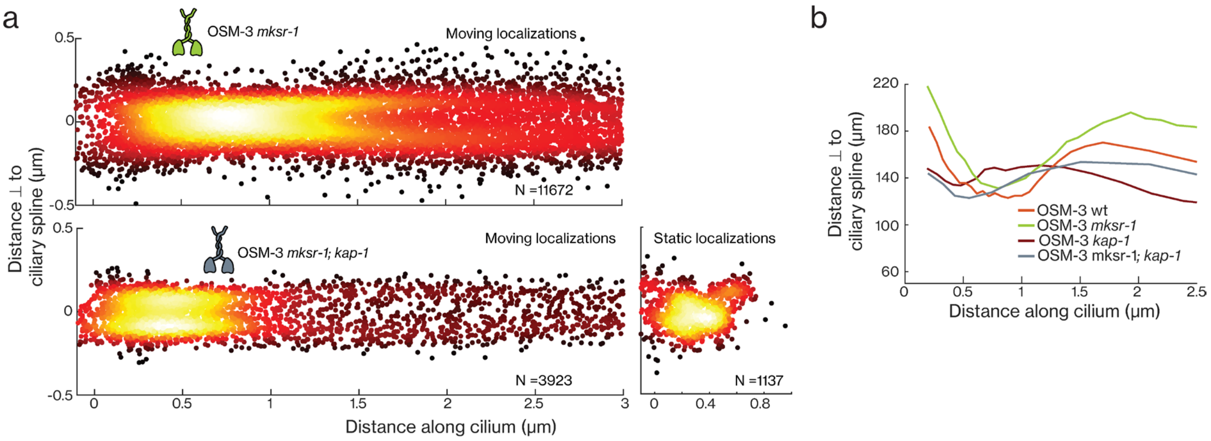

Supplementary Fig. 8: Structural details obtained from tracking single OSM-3 *mksr-1* and OSM-3 *mksr-1; kap-1* molecules entering cilia. **(a)** Super-resolution map of single-molecule localizations obtained from 428 OSM-3 *mksr-1* and 173 OSM-3 *mksr-1; kap-1* tracks. Upper panel: N = 11672 moving localizations of OSM-3 *mskr-1*. Lower left panel: N = 3923 moving localizations of OSM-3 *mksr-1; kap-1*. Lower right panel: N = 1137 static localizations of OSM-3 *mksr-1; kap-1*. **(b)** Width of the distribution of single-molecule localizations with respect to the ciliary spline, along the ciliary length, for OSM-3 in wild type (orange), OSM-3 *kap-1* (brown), OSM-3 *mksr-1* (green), OSM-3 *mksr-1; kap-1* (grey).

Supplementary Table:

Strain	Genotype	Publication	Name used
EJP13	*Kap-1(ok676) III; vuaSi1 [pBP20; Pkap-1::kap-1::eGFP; cb-unc-119(+)] IV*	(Prevo et al., 2015)	kinesin-II
EJP212	*vuaSi26 [Pxbx-1::xbx-1::EGFP; cb-unc119(+)] I; vuaSi2 [Posm-3::osm3::mCherry; cb-unc-119(+)] II; osm3(p802) IV; xbx-1(ok279) V*	(Mijalkovic et al., 2017)	IFT-dynein and OSM-3
EJP401	*vuaSi401 [pSA401; Ptbb-4::tbb-4::EGFP; cb-unc-119(+)] I; unc-119(ed3) III*	(Mijalkovic et al., 2018)	tubulin
EJP22	*vuaSi2 [pBP22; Posm-3::osm-3::mCherry; cb-unc-119(+)] II; kap-1(ok676)III; osm-3(p802) IV*	(Prevo et al., 2015)	OSM-3 *kap-1*
EJP213	*vuaSi26 [Pxbx-1::xbx-1::EGFP; cb-unc119(+)] I; vuaSi2 [Posm-3::osm3::mCherry; cb-unc-119(+)] II; kap1(ok676) III; osm-3(p802) IV; xbx1(ok279) V*	(Mijalkovic et al., 2017)	IFT-dynein *kap-1*
EJP41	*vuaSi10 [pBP33; Pkap-1::kap-1::eGFP; cb-unc-119(+)] I; osm-3(p802) IV*	(Prevo et al., 2015)	kinesin-II *osm-3*
EJP72	*vuaSi2 [pBP22; Posm-3::osm-3::mCherry; cb-unc-119(+)] II; kap-1(ok676)III; osm-3(p802) IV; mksr-1(ok2092) X*	This study	OSM-3 *mksr-1*
EJP64	*Kap-1(ok676) III; vuaSi10 [pBP33; Pkap-1::kap-1::eGFP; cb-unc-119(+)] I; mksr-1(ok2092) X*	(Prevo et al., 2015)	kinesin-II *mksr-1*
EJP73	*vuaSi2 [pBP22; Posm-3::osm-3::mCherry; cb-unc-119(+)] II; kap-1(ok676)III; osm-3(p802) IV; mksr-1(ok2092) X*	(Prevo et al., 2015)	OSM-3 *kap-1; mksr-1*

Supplementary Table 1: *C. elegans* strains used in this study. Short notation is used throughout the main text and figures to increase readability.
