# Supplementary material for: IFT cargo and motors associate sequentially with IFT trains to enter cilia of C. elegans
Source: Nat Commun. 2024 Apr 24;15:3456. doi: 10.1038/s41467-024-47807-2 (PMC11043347; doi:10.1038/s41467-024-47807-2)
Supplement: Supplementary file 3 — Description of Additional Supplementary Files [file 41467_2024_47807_MOESM3_ESM.pdf]

Description of Additional Supplementary Files:

IFT cargo and motors associate sequentially with IFT trains to enter cilia of *C. elegans*

Aniruddha Mitra[1,2], Elizaveta Loseva[1] and Erwin J.G. Peterman [1,*]

[1] Department of Physics and Astronomy and LaserLaB, Vrije Universiteit Amsterdam, Netherlands
[2] Present address: Cell Biology, Neurobiology and Biophysics, Department of Biology, Faculty of Science, Utrecht University, The Netherlands.
* Correspondence: e.j.g.peterman@vu.nl

Supplementary Movie 1: Single-molecule imaging of IFT dynein in the cilia of *C. elegans* either using the full excitation beam or using SWIM. XBX-1::eGFP single-molecule dynamics in the cilia of PHA/PHB neurons, imaged for 25 min at the acquisition rate 6.6 fps, with the aperture open (beam width ~30 µm; left panel) and aperture closed (beam width ~10 µm; right panel). The videos are displayed from 20 min onwards. Scale bar is 2 µm and time is indicated in min:sec.

Supplementary Movie 2: Single-molecule imaging of IFT components in wild-type cilia. Single-molecule dynamics of kinesin-II (KAP-1::eGFP; **a**), OSM-3 (OSM-3::mCherry; **b**), IFT-dynein (XBX-1::eGFP; **c**) and tubulin (TBB-4::eGFP; **d**), imaged using SWIM. Scale bar is 2 µm and time is indicated in min:sec.

Supplementary Movie 3: Fast single-molecule imaging of IFT components in wild-type cilia. (a) Example molecules of kinesin-II moving diffusively in the PCMC before docking at the ciliary base and entering the cilium. **(b)** Example kinesin-II molecule docking and undocking at the ciliary base, with diffusive phases in between. **(c)** Example IFT-dynein molecules moving diffusively in the PCMC before docking at the ciliary base. **(d)** Example OSM-3 molecule docking on a moving train at the ciliary base and entering further into the cilium. Image acquisition was performed at ~60 fps for kinesin-II (KAP-1::eGFP) and IFT-dynein (XBX-1::eGFP) and at ~31 fps for OSM-3 (OSM-3::mCherry). For each video segment the corresponding maximum intensity projection (obtained from the entire 3-5 min long video acquisition) is provided in the upper panel to visualize the cilia pair. The ciliary base where the example molecules dock is indicated by white arrowheads. The kymographs corresponding to the video segments are shown in Supplementary Fig. 4e-4h. Scale bar is 2 µm.

Supplementary Movie 4: Single-molecule imaging of IFT components in kinesin-II loss-of-function mutants. Single-molecule dynamics of OSM-3 (OSM-3::mCherry; left panel) and IFT-dynein (XBX-1::eGFP; right panel) in *kap-1* mutant worms, imaged using SWIM. Scale bar is 2 µm and time is indicated in min:sec.

Supplementary Movie 5: Single-molecule imaging of OSM-3 in *mksr-1* and mksr-1; kap-1 mutants. Single-molecule dynamics of OSM-3 in mksr-*1* mutant worms (left panel) and *mksr-1; kap-1* mutant worms (right panel), imaged using SWIM. Scale bar is 2 µm and time is indicated in min:sec.

Supplementary Movie 6: Single-molecule imaging of IFT-dynein in amphid cilia. Maximum intensity projection (left panel) and the corresponding video (center panel) showing ensemble IFT-dynein dynamics in an amphid cilia bundle. Imaging acquisition was performed at 1% of the maximal laser intensity. Right panel: Single-molecule dynamics of IFT-dynein in the same amphid cilia, imaged using SWIM, at the maximal laser intensity. Individual IFT-dynein molecules, diffusive at the dendrites, dock stochastically at the base of several cilia, pausing briefly, before entering the cilia. Scale bar is 2 µm.
