# Supplementary material for: IFT cargo and motors associate sequentially with IFT trains to enter cilia of C. elegans
Source: Nat Commun. 2024 Apr 24;15:3456. doi: 10.1038/s41467-024-47807-2 (PMC11043347; doi:10.1038/s41467-024-47807-2)
Supplement: Supplementary file 10 — Reporting Summary [file 41467_2024_47807_MOESM10_ESM.pdf]

nature portfolio

Corresponding author(s): Erwin J. G. Peterman

Last updated by author(s): March 18 2024

Reporting Summary

Nature Portfolio wishes to improve the reproducibility of the work that we publish. This form provides structure for consistency and transparency in reporting. For further information on Nature Portfolio policies, see our Editorial Policies and the Editorial Policy Checklist.

Statistics

For all statistical analyses, confirm that the following items are present in the figure legend, table legend, main text, or Methods section.

n/a	Confirmed	
[ ]	[x]	The exact sample size (*n*) for each experimental group/condition, given as a discrete number and unit of measurement
[ ]	[x]	A statement on whether measurements were taken from distinct samples or whether the same sample was measured repeatedly
[ ]	[x]	The statistical test(s) used AND whether they are one- or two-sided<br>*Only common tests should be described solely by name; describe more complex techniques in the Methods section.*
[x]	[ ]	A description of all covariates tested
[x]	[ ]	A description of any assumptions or corrections, such as tests of normality and adjustment for multiple comparisons
[ ]	[x]	A full description of the statistical parameters including central tendency (e.g. means) or other basic estimates (e.g. regression coefficient) AND variation (e.g. standard deviation) or associated estimates of uncertainty (e.g. confidence intervals)
[x]	[ ]	For null hypothesis testing, the test statistic (e.g. *F*, *t*, *r*) with confidence intervals, effect sizes, degrees of freedom and *P* value noted<br>*Give P values as exact values whenever suitable.*
[x]	[ ]	For Bayesian analysis, information on the choice of priors and Markov chain Monte Carlo settings
[x]	[ ]	For hierarchical and complex designs, identification of the appropriate level for tests and full reporting of outcomes
[x]	[ ]	Estimates of effect sizes (e.g. Cohen's *d*, Pearson's *r*), indicating how they were calculated

Our web collection on statistics for biologists contains articles on many of the points above.

Software and code

Policy information about availability of computer code

Data collection	All microscopy data was acquired using Micromanager (version 1.4)
Data analysis	Particle tracking was performed using a MATLAB-based software, FIESTA (version 1.6.0) (Ruhnow et al., 2011).<br>Fiji (ImageJ) v.2.0.0 was used to obtain intensity information for FRAP experiments and measuring bleaching rate and for generating kymographs.<br>Custom MATLAB codes (The Math Works, Inc., R2021a) were written for data analysis, visualization of data and numerical simulations, which are available on DataverseNL: https://doi.org/10.34894/EBPWXD

For manuscripts utilizing custom algorithms or software that are central to the research but not yet described in published literature, software must be made available to editors and reviewers. We strongly encourage code deposition in a community repository (e.g. GitHub). See the Nature Portfolio guidelines for submitting code & software for further information.

Data

Policy information about availability of data

All manuscripts must include a data availability statement. This statement should provide the following information, where applicable:

- Accession codes, unique identifiers, or web links for publicly available datasets
- A description of any restrictions on data availability
- For clinical datasets or third party data, please ensure that the statement adheres to our policy

The underlying experimental data in this study can be found in the source data file associated with the manuscript. Further, the same data along with scripts (written on MATLAB) to visualize the data as displayed in the figures are available on DataverseNL: https://doi.org/10.34894/EBPWXD

Research involving human participants, their data, or biological material

Policy information about studies with human participants or human data. See also policy information about sex, gender (identity/presentation), and sexual orientation and race, ethnicity and racism.

Reporting on sex and gender	N.A.
Reporting on race, ethnicity, or other socially relevant groupings	N.A.
Population characteristics	N.A.
Recruitment	N.A.
Ethics oversight	N.A.

Note that full information on the approval of the study protocol must also be provided in the manuscript.

Field-specific reporting

Please select the one below that is the best fit for your research. If you are not sure, read the appropriate sections before making your selection.

☒ Life sciences ☐ Behavioural & social sciences ☐ Ecological, evolutionary & environmental sciences

For a reference copy of the document with all sections, see nature.com/documents/nr-reporting-summary-flat.pdf

Life sciences study design

All studies must disclose on these points even when the disclosure is negative.

Sample size	No calculations were done to predetermine sample size. Suitable sample sizes were determined based on previous experience and based on published literature in the field. A minimum of 7 organisms were imaged for every C. elegans worm strain. The overview of the sample sizes, such as number of organisms (C. elegans), cilia number, tracked events and tracked localizations is provided in Table 1. Further, sample sizes are also mentioned in Figures and Figure captions.
Data exclusions	For analysis of tracks of single-molecule events, only tracks longer than 12 frames were used. Also other, clearly defined, factors were taken into account to disregard certain tracks (or frames from a track). These factors are described in detail in the Image analysis section in the Methods section of the manuscript.
Replication	Single molecule imaging of each C. elegans strain were repeated in at least two independent imaging sessions.
Randomization	All organisms of a given C. elegans strain were genetically identical and they were randomly selected for imaging.
Blinding	No blinding was performed in this study. The phenotypes of the IFT components imaged in the different C elegans strains were readily observable from the data, consequently blinding would not be effective.

Reporting for specific materials, systems and methods

We require information from authors about some types of materials, experimental systems and methods used in many studies. Here, indicate whether each material, system or method listed is relevant to your study. If you are not sure if a list item applies to your research, read the appropriate section before selecting a response.

Materials & experimental systems

n/a	Involved in the study
☒	☐ Antibodies
☒	☐ Eukaryotic cell lines
☒	☐ Palaeontology and archaeology
☐	☒ Animals and other organisms
☒	☐ Clinical data
☒	☐ Dual use research of concern
☒	☐ Plants

Methods

n/a	Involved in the study
☒	☐ ChIP-seq
☒	☐ Flow cytometry
☒	☐ MRI-based neuroimaging

Animals and other research organisms

Policy information about studies involving animals; ARRIVE guidelines recommended for reporting animal research, and Sex and Gender in Research

Laboratory animals	C. elegans strains used in this study are mentioned in Supplementary Table 1. Imaging was performed only on young adult hermaphrodite nematodes (with approximately 5-10 eggs in the body)
Wild animals	No wild animals were used in this study.
Reporting on sex	Only hermaphrodite nematodes were imaged in this study.
Field-collected samples	No field collected samples were used in the study.
Ethics oversight	No ethical approval was required for this study.

Note that full information on the approval of the study protocol must also be provided in the manuscript.

Plants

Seed stocks	N.A.
Novel plant genotypes	N.A.
Authentication	N.A.
